# Prevention of hyperglycemia-induced myocardial apoptosis by gene silencing of Toll-like receptor-4

**DOI:** 10.1186/1479-5876-8-133

**Published:** 2010-12-15

**Authors:** Yuwei Zhang, Tianqing Peng, Huaqing Zhu, Xiufen Zheng, Xusheng Zhang, Nan Jiang, Xiaoshu Cheng, Xiaoyan Lai, Aminah Shunnar, Manpreet Singh, Neil Riordan, Vladimir Bogin, Nanwei Tong, Wei-Ping Min

**Affiliations:** 1 Department of Endocrinology, West China Hospital of Sichuan University, Chengdu, China; 2 Departments of Surgery, Pathology, Medicine, Oncology, University of Western Ontario, London, Ontario, Canada; 3 Lawson Health Research Institute, London Health Sciences Centre, London, Ontario, Canada; 4 Nanchang University Second Affiliated Hospital, Nanchang, China; 5 Medistem Panama City of Knowledge, Clayton, Republic of Panama; 6 Medistem Inc, San Diego, CA, USA

## Abstract

**Background:**

Apoptosis is an early event involved in cardiomyopathy associated with diabetes mellitus. Toll-like receptor (TLR) signaling triggers cell apoptosis through multiple mechanisms. Up-regulation of TLR4 expression has been shown in diabetic mice. This study aimed to delineate the role of TLR4 in myocardial apoptosis, and to block this process through gene silencing of TLR4 in the myocardia of diabetic mice.

**Methods:**

Diabetes was induced in C57/BL6 mice by the injection of streptozotocin. Diabetic mice were treated with 50 μg of TLR4 siRNA or scrambled siRNA as control. Myocardial apoptosis was determined by TUNEL assay.

**Results:**

After 7 days of hyperglycemia, the level of TLR4 mRNA in myocardial tissue was significantly elevated. Treatment of TLR4 siRNA knocked down gene expression as well as diminished its elevation in diabetic mice. Apoptosis was evident in cardiac tissues of diabetic mice as detected by a TUNEL assay. In contrast, treatment with TLR4 siRNA minimized apoptosis in myocardial tissues. Mechanistically, caspase-3 activation was significantly inhibited in mice that were treated with TLR4 siRNA, but not in mice treated with control siRNA. Additionally, gene silencing of TLR4 resulted in suppression of apoptotic cascades, such as Fas and caspase-3 gene expression. TLR4 deficiency resulted in inhibition of reactive oxygen species (ROS) production and NADPH oxidase activity, suggesting suppression of hyperglycemia-induced apoptosis by TLR4 is associated with attenuation of oxidative stress to the cardiomyocytes.

**Conclusions:**

In summary, we present novel evidence that TLR4 plays a critical role in cardiac apoptosis. This is the first demonstration of the prevention of cardiac apoptosis in diabetic mice through silencing of the TLR4 gene.

## Introduction

Hyperglycemia is the underlying abnormality characterizing the diabetic condition. Chronic hyperglycemia introduces a plethora of complications such as cardiovascular disease, which is the most frequent cause of death in the diabetic population [1]. Diabetic patients have a poorer prognosis post-myocardial infarction as well as an increased risk of subsequent heart failure [2,3]. Studies have shown hyperglycemic patients hospitalized with acute coronary syndromes also have higher mortality rates [4]. A key pathological consequence of sustained hyperglycemia is the induction of cardiomyocyte apoptosis reported in both diabetic patients and animal models of diabetes [5]. Cardiomyocyte apoptosis causes a loss of contractile units which reduces organ function and provokes cardiac remodeling, which is associated with hypertrophy of viable cardiomyocytes [5-8]. As such, should myocardial apoptosis be inhibited, one would expect to prevent or slow the development of heart failure. Yet, the means by which hyperglycemia induces apoptosis in cardiomyocytes have not been fully understood.

Toll-like receptor 4 (TLR4) is a key proximal signaling receptor responsible for initiating the innate immune response. TLR4 recognizes pathogen-associated molecular patterns and plays a vital role in myocardial dysfunction during bacterial sepsis [9] and pressure overload-induced cardiac hypertrophy. TLR4 expression is elevated in failing and ischemic human hearts as well as in animal models of myocardial ischemia [10,11]. In addition, recent studies suggest TLR4 may trigger apoptosis of cardiomyocytes in conditions of cardiac inflammation and oxidative stress [12]. Studies have also shown that TLR4 is increased in diabetic mice, however, the role of TLR4 in hyperglycemia-induced myocardial apoptosis has not been elucidated. In this study, we initially investigated the role of TLR4 on apoptosis in cardiomyocytes under hyperglycemic conditions. Subsequently, we explored the intervention of apoptosis in cardiomyocytes through RNA interference (RNAi) using small interfering RNA (siRNA) specific to TLR4 gene. We found that TLR4 was up-regulated in the myocardia of STZ-treated diabetic mice (STZ mice), which displayed increased expression of apoptotic genes such as Fas and caspase-3. Treatment with TLR4 siRNA attenuated apoptosis as well suppressed ROS production and NADPH oxidase activity.

## Materials and methods

### Animals

C57/BL6 mice were purchased from The Jackson Laboratory (Bar Harbor, ME, USA). All mice were male and 6-8 weeks old. All experimental procedures were approved by the Animal Use Sub-committee at the University of Western Ontario, Canada, in accordance with the Guide for the Care and Use on Animals Committee Guidelines.

### Hyperglycemic mouse model

Adult male mice (6-8 weeks old) were intraperitoneally injected with a single dose of streptozotocin (STZ) at 150 mg/kg body weight, dissolved in 10 mM sodium citrate buffer (pH 4.5). On day 3 after STZ treatment, whole blood was obtained from the mouse tail vein and random glucose levels were measured using the OneTouch Ultra 2 blood glucose monitoring system (LifeScan, Mountainview, CA). For the present study, hyperglycemia is defined as a blood glucose measurement of 20 mM or higher. Citrate buffer-treated mice were used as a normoglycemic control (blood glucose <12 mM).

### siRNA expression vectors

Three target sequences of TLR4 gene were selected. The oligonucleotides containing sequences specific for TLR4 (5'-GATCCCGTATTAGGAACTACCTCTATGCTTGATATC CGGCATAGAGGTAGTTCCTAATATTTTTTCCAAA-3' and 5'-AGCTTTTGGAAAAA ATATTAGGAACTACCTCTATGCCGGATATCAAGCATAGAGGTAGTTCCTAATA CGG-3'; 5'-GATCCCGTTGAAACTGCAATCAAGAGTGTTGATATCCGCACTCTTG ATTGCAGTTTCAATTTTTTCCAAA-3'and 5'-AGCTTTTGGAAAAAATTGAAACT GCAATCAAGAGTGCGGATATCAACACTCTTGATTGCAGTTTCAACGG-3'; 5'-GATCCCATTCGCCAAGCAATGGAACTTGATATCCGGTTCCATTGCTTGGCGAA TTTTTTTCCAAA-3'and 5'-AGCTTTTGGAAAAAAATTCGCCAAGCAATGGAACCG GATATCAAGTTCCATTGCTTGGCGAATGG-3') were synthesized and annealed. A TLR4-siRNA expression vector that expresses hairpin shRNA under the control of the mouse U6 promoter was constructed. A pair of annealed DNA oligonucleotides were inserted into a pRNAT-U6.1/Neo shRNA expression vector that had been digested with BamHI and HindIII (Genescript, Piscataway, NJ, USA). The plasmid was suspended in water and stored at -80°C until use.

### Treatment of TLR4 siRNA

TLR4 siRNA or scrambled siRNA (50 μg) was mixed with 40 μl of transfection reagent NANOPARTICLE (Altogen Biosystems, Las Vegas, NV, USA) with total volume of 500 μl of 5% glucose (W/V), as per the manufacturer's instruction. The siRNA mixture was intravenously injected into the C57/BL6 mouse via the tail vein.

### Real-time PCR

Total RNA was isolated from heart tissues using Trizol reagent (Invitrogen) according to the manufacturer's protocol. The RNA was subsequently reverse-transcribed using an oligo-(dT) primer and reverse transcriptase (Invitrogen). Primers used for the amplification of murine TLR4, Fas, caspase-3 and an internal loading control, glyceraldehyde-3-phosphate dehydrogenase (GAPDH) were respectively, as follows: TLR4, sense 5'-CACTGTTCTTCTCCTGCCTGAC-3' (forward), and 5'-CCTGGGGAAAAACTCT GGATAG-3' (reverse); Fas, 5'-CAGAAATCGCCTATGGTTGTTG-3' (forward), and 5'-GCT CAGCTGTGTCTTGGATGC-3' (reverse); caspase-3, 5'-TGACCATGGAGAACAACAAA ACCT-3' (forward), and 5'-TCCGTACCAGAGCGAGATGACA-3' (reverse); and GAPDH, 5'-TGATGACATCAAGAAGGTGGTGAA-3' (forward) and 5'-TGGGATGGAAATTGT GAGGGAGAT-3' (reverse).

Real-time PCR reactions were performed using SYBR Green PCR Master mix (Stratagene) and 80 nM of gene-specific forward and reverse primers as described above. The PCR reaction conditions were 95°C for 10 min, 95°C for 30 sec, 58°C for one min and 72°C for 30 sec (40 cycles). Amplification was performed according to the manufacturer's cycling protocol and done in triplicate. Gene expression was calculated as 2^-ΔΔ(Ct) ^[13], where Ct is cycle threshold, ΔΔ(Ct) = sample 1Δ(Ct) -sample 2Δ(Ct); Δ(Ct) = *GAPDH *(Ct) - testing gene (Ct). Data was analyzed using MX4000 (Stratagene), Microsoft Excel 2003, and GraphPad Prism software.

### In situ detection of apoptotic cells

Apoptosis in heart tissue was detected using the ApopTag in situ apoptosis detection kit (Qbiogene, Illkirch, France), as specified by the manufacturer. Briefly, paraffin embedded sections were deparaffinized and pre-treated with proteinase K (20 μg/ml) for 15 min. Equilibration buffer was added directly onto the specimen, after which terminal deoxynucleotidyl transferase (TdT) enzyme in reaction buffer was added for 1 h at 37°C. Sections were washed in Stop/Wash buffer for 10 min. After incubating with anti-digoxigenin peroxidase conjugate for 30 min, the peroxidase substrate was added to develop color. The samples were washed with PBS and observed under a microscope in a blinded fashion, and the proportion of cardiac cells undergoing apoptosis was calculated.

### Caspase-3 Activity

Caspase-3 activity in myocardial tissues was measured by using a caspase-3 fluorescent assay kit (BIOMOL Research Laboratory), as described previously [14]. Briefly, hearts from diabetic mice were homogenized, and protein concentration was determined using the Bradford method. Samples in duplicates were incubated with caspase-3 substrate Ac-DEVD-AMC or Ac-DEVD-AMC plus inhibitor AC-DEVD-CHO at 37°C for 2 h before measurements were made by a fluorescent spectrophotometer (excitation at 380 nm, emission at 405 nm). Signals from inhibitor-treated samples served as background.

### NADPH oxidase activity assay

NADPH oxidase activity was assessed in cell lysates by lucigenin-enhanced chemiluminescence (20 μg of protein, 100 μM NADPH, 5 μM lucigenin) with a multilabel counter (Victor3 Wallac), as described previously [15].

### Intracellular ROS measurement

The formation of ROS was measured using the ROS-sensitive dye, 2,7-dichlorodihydro-fluorescein diacetate (DCF-DA, Invitrogen), as an indicator. The assay was performed on freshly dissected heart tissues. Samples (50 μg proteins) were incubated with 10 μl of DCF-DA (10 μM) for 3 h at 37°C. The fluorescent product formed was quantified by spectrofluorometer at the 485/525 nm. Changes in fluorescence were expressed as an arbitrary unit.

### Statistical analysis

Data were expressed as the mean ± SD. Differences between two groups were compared by unpaired Student's *t*-test. For multi-group comparison, data were compared using a one-way analysis of variance (ANOVA) followed by the Newman-Keuls test analysis. Differences for the value of p < 0.05 were considered significant.

## Results

### 1. Up-regulation of TLR4 and apoptosis in myocardial tissue of STZ mice

**Figure 1:**
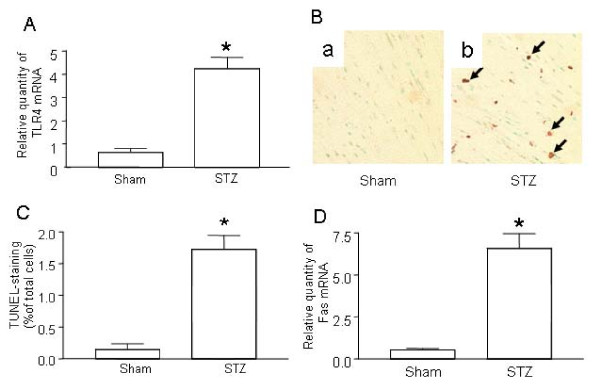
**Up-regulation of TLR4 and increased apoptosis in the hearts of STZ mice**. ***(A) ***TLR4 expression in the hearts of STZ mice. Injection of STZ induced Type I diabetes as described in Materials and Methods. Control mice were injected with the same volume of sodium citrate buffer (Sham). On day 7 after STZ treatment, the hearts from diabetic mice (n = 6) and sham mice (n = 6) were retrieved. Total mRNA was extracted and used to detect the TLR4 transcripts by qPCR. ***(B) ***Determination of in situ apoptotic cells in myocardia. Apoptosis in sham-treated mice and STZ-treated diabetic mice was detected by TUNEL assay. Representative photomicrographs of TUNEL staining in cardiomyocytes are shown in yellow-blown signal (arrows) from (a) sham treated mice (n = 6) or (b) STZ-treated diabetic mice (n = 6). ***(C) ***Quantification of TUNEL positive cardiomyocytes. ***(D) ***Fas expression in the hearts of STZ mice. Diabetes was induced by STZ injection as described in Materials and Methods. On day 7 after STZ treatment, the hearts from diabetic mice (n = 6) and sham mice (n = 6) were retrieved. Total mRNA was extracted and used to detect the Fas transcripts by qPCR. Mean ± SD are shown in A, C and D, and are representative of 3 experiments; (∗) Statistical significance when compared with sham treated mice and STZ-treated diabetic mice was denoted at p < 0.05.

Although TLRs are reportedly up-regulated in cardiomyocytes of diabetic patients [11], it is unclear whether TLRs play a role in the promotion of diabetes in the initial stages of disease or if their up-regulation is a consequence of stimulation from hyperglycemia. To clarify this, we measured TLR4 levels in mice in the early stages of diabetes. After treatment with STZ, C57/BL6 mice developed diabetes as evidenced by hyperglycemia (data not shown). Significantly increased TLR4 was detected in the myocardial tissue of STZ-mice as early as 3 days after the appearance of hyperglycemia (Figure 1A).

We and others have previously demonstrated that hyperglycemia is capable of inducing apoptosis in cardiomyocytes [16-18]. Apoptosis is one of the earliest indicators of cardiomyopathy in the diabetic heart and accordingly, we measured apoptosis in STZ-treated mice. Seven days after STZ treatment, substantial apoptosis was detected in myocardial tissue (Figure 1B). Additionally, Fas expression was significantly increased in STZ-treated mice compared to control littermates (Figure 1D).

### 2. Prevention of hyperglycemia-induced apoptosis in myocardial tissue by gene silencing of TLR4

**Figure 2:**
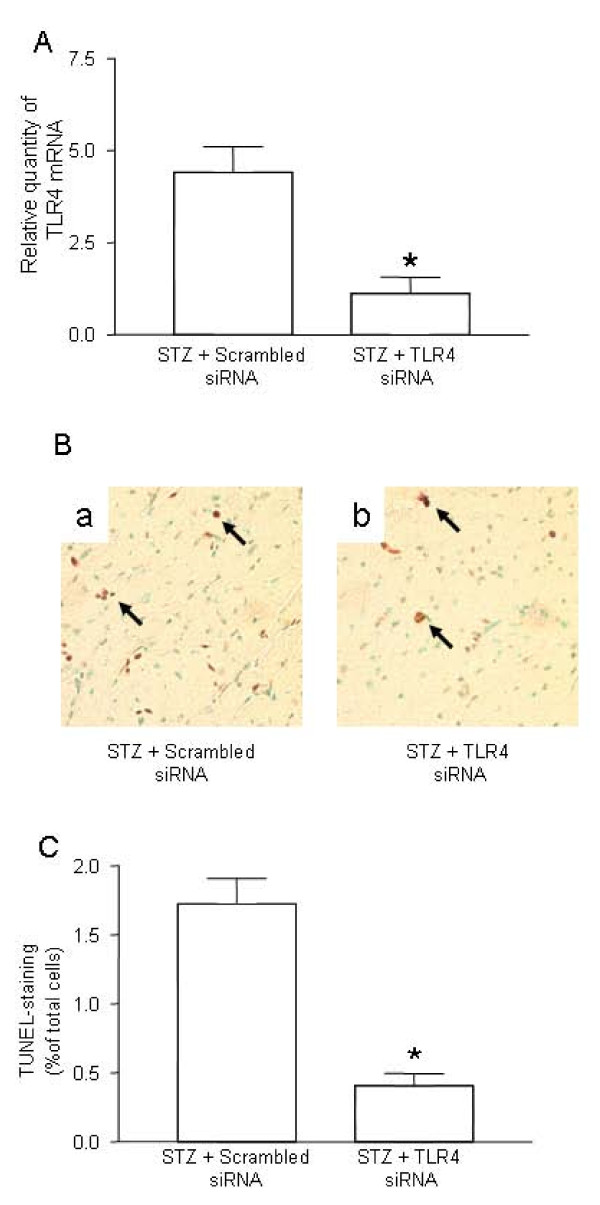
**Suppression of TLR4 and prevention of apoptosis by gene silencing of TLR4**. ***(A) ***Suppression of TLR4 expression in the heart of STZ mice treated with TLR4 siRNA. Diabetes was induced by STZ injection as described in Materials and Methods. On day -1 (the day before STZ treatment), mice were intravenously injected with 5 μg of TLR4 siRNA or scrambled control siRNA, along with NANOPARTICLE. On day 7 after STZ treatment, the hearts from the mice treated with TLR4 siRNA (n = 6) or scrambled siRNA (n = 6) were retrieved. Total mRNA was extracted and used to detect the TLR4 transcripts by qPCR. The relative quantity of TLR4 mRNA was expressed as mean ± SD. (∗) Statistical significance when compared with scrambled siRNA treated mice was denoted as p < 0.05. ***(B) ***Attenuation of apoptotic cells in cardiomyocyte by TLR4 siRNA. Apoptosis in the diabetic mice treated with control siRNA (n = 6) and TLR4 siRNA (n = 6) was detected by TUNEL assay. Representatives of TUNEL staining in cardiomyocytes were shown in yellow-blown signal (arrows) from the mice treated with scrambled siRNA (a) or TLR4 siRNA (b). ***(C) ***Quantification of TUNEL positive cardiomyocytes. Data shown are representative of 3 experiments.

Accumulating evidence suggests that activation of the TLR4 pathway is associated with myocardial apoptosis [12]. We explored whether knockdown of TLR4 may suppress apoptosis of cardiomyocytes in STZ-mice. First, we validated *in vivo *gene silencing of TLR4 siRNA in myocardial tissue. After infusion of TLR4 siRNA, the TLR4 mRNA level was decreased by 75%, as comparing with the mice treated with scrambled control siRNA (Figure 2A), indicative of successful knockdown in the heart *in vivo*. Treatment with TLR4 siRNA did not affect the level of blood glucose in diabetic mice (Data not shown).

Next, we examined whether gene knockdown of TLR4 has a therapeutic effect on the prevention of myocardial apoptosis in diabetic mice. As shown in Figure 2B, apoptosis, as detected by the TUNEL assay, was remarkably attenuated in mice treated with TLR4 siRNA compared with scrambled siRNA.

### 3. Inhibition of caspase-3 in myocardia after gene silencing of TLR4

**Figure 3:**
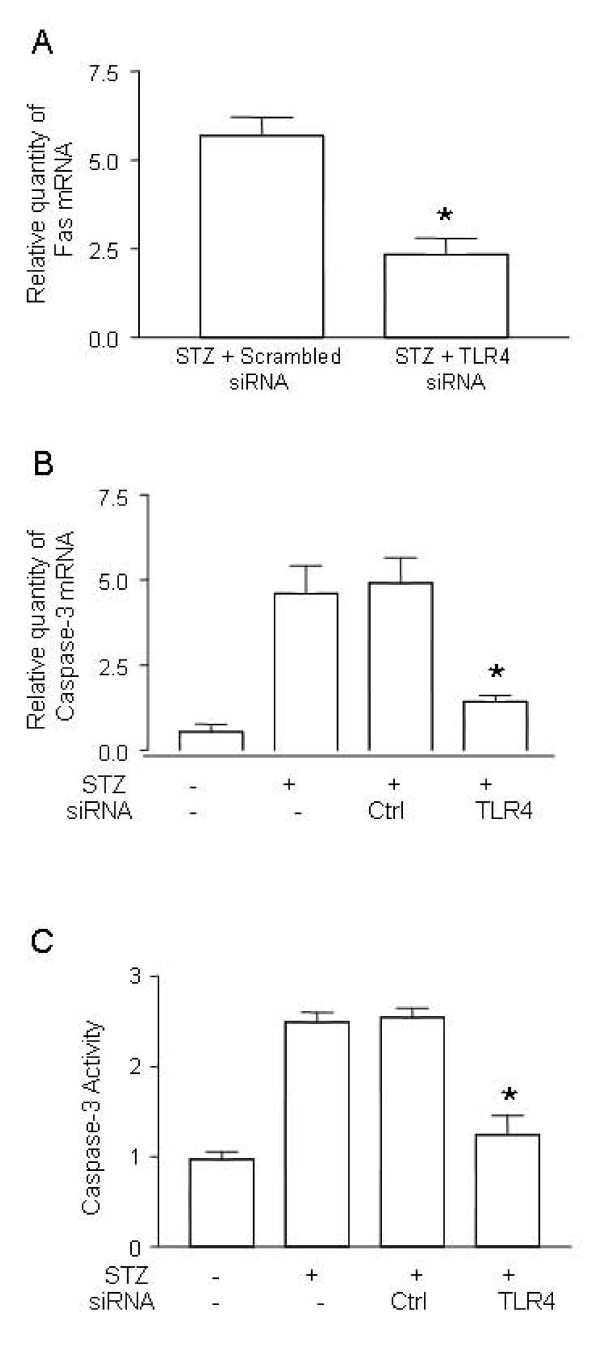
**Inhibition of caspase-3 after gene silencing of TLR4**. ***(A) ***Suppression of Fas expression in the hearts of STZ mice treated with TLR4 siRNA. Diabetes was induced by STZ injection as described in Materials and Methods. Diabetic mice were treated with TLR4 siRNA (n = 6) and scrambled control siRNA (n = 6) as described in Figure 2. On day 7 after STZ treatment, the hearts from mice treated with TLR4 siRNA or scrambled siRNA were retrieved. Total mRNA was extracted and used to detect Fas transcripts by qPCR. ***(B) ***Suppression of caspase-3 expression in the heart of STZ mice treated with TLR4 siRNA. Diabetic mice were treated with TLR4 siRNA (n = 6) and scrambled control siRNA (n = 6) as described above. The expression of caspase-3 transcripts was detected by qPCR. *(**C) ***Inhibition of caspase-3 activity in the heart of STZ mice treated with TLR4 siRNA. Diabetic mice were treated with TLR4 siRNA (n = 6) and scrambled control siRNA (n = 6) as described above. On day 7 after STZ treatment, the hearts from the mice treated with TLR4 siRNA or scrambled siRNA were retrieved, the protein was prepared and the caspase-3 activity was determined as described in Methods and Materials. Relative quantity of TLR4 mRNA and caspase-3 activity was expressed as mean ± SD. (∗) Statistical significance when compared with scrambled siRNA treated mice was denoted as p < 0.05. Data shown are representative of 3 experiments.

To further confirm the Fas-FasL pathway is involved in apoptosis of cardiomyocytes, we measured the expression of Fas in the myocardial tissue of STZ mice. Treatment of TLR4 siRNA resulted in the suppression of Fas expression (Figure 3A).

To understand the involvement of pro-apoptotic caspases, we determined caspase-3 levels in myocardial tissue. Sham-treated control mice only expressed low level of caspase-3 while in heart tissue of STZ-treated mice, hyperglycemia was shown to up-regulate caspase-3 expression dramatically (Figure 3B). Treatment of control siRNA did not alter the level of caspase-3; however, treatment of TLR4 siRNA effectively reversed up-regulation of caspase-3 (Figure 3B).

To confirm caspase-3 gene suppression influences its biological function in the apoptotic pathway, we measured caspase-3 activity in the myocardial tissue. Caspase-3 activation was remarkably inhibited in mice treated with TLR4 siRNA but not in mice treated with scrambled siRNA or non-treated diabetic mice (Figure 3C).

### 4. Attenuation of ROS production in myocardia after gene silencing of TLR4

**Figure 4:**
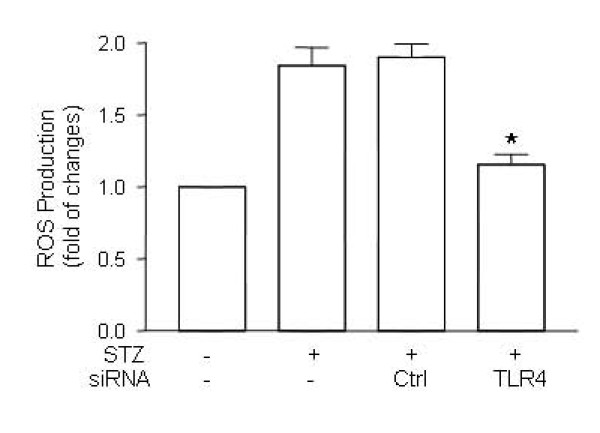
**Inhibition of ROS production in TLR4-silenced STZ mice**. Diabetes was induced by STZ injection as described in Materials and Methods. Diabetic mice were treated with TLR4 siRNA and scrambled control siRNA as described in Figure 2. On day 7 after STZ treatment, the hearts from mice treated with TLR4 siRNA (n = 6) or scrambled siRNA (n = 6) were retrieved, the protein was prepared and the ROS production was determined as described in Methods and Materials. Data are representative of 3 repeated experiments, and are shown as mean ± SD. (∗) Statistical significance when compared with scrambled siRNA treated mice was denoted as p < 0.05.

It has been demonstrated that hyperglycemia may stimulate the production of reactive oxygen species (ROS) which in turn induces apoptosis in the diabetic heart [17,19]. We measured ROS levels in the myocardia of STZ-treated mice in order to examine the contribution of ROS production to apoptosis and found that ROS production was increased in mice with hyperglycemia (Figure 4). While the treatment of scrambled siRNA did not change the production of ROS in STZ mice, treatment of TLR4 siRNA resulted in significant decrease in ROS production in the diabetic heart (Figure 4).

### 5. Suppression of NADPH oxidase activity in TLR4-silenced STZ mice

**Figure 5:**
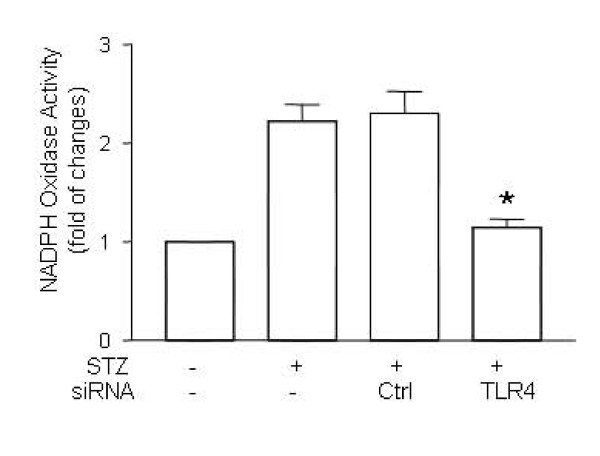
**Suppression of NADPH oxidase activity in TLR4-silenced STZ mice**. Diabetes was induced by STZ injection as described in Materials and Methods. Diabetic mice were treated with TLR4 siRNA and scrambled control siRNA as described in Figure 2. On day 7 after STZ treatment, the hearts from mice treated with TLR4 siRNA (n = 6) or scrambled siRNA (n = 6) were retrieved, the protein was prepared and the NADPH oxidase activity was determined as described in Methods and Materials. Data are representative of 3 repeated experiments, and are shown as mean ± SD. (∗) Statistical significance when compared with scrambled siRNA treated mice was denoted as p < 0.05.

It has been recently reported that myocardial NADPH oxidase activity is up-regulated in diabetes [17,20]. Additionally, accumulating evidence suggests that hyperglycemia activates NADPH oxidase in cardiomyocytes [21]. Our previous study showed that NADPH oxidase contributed to hyperglycemia-induced apoptosis [17]. To explore the role of NADPH in TLR-induced myocardial apoptosis, we measured NADPH oxidase activity. As shown in Figure 5, NADPH oxidase activity in STZ-mice was significantly increased. Treatment with TLR4 siRNA suppressed up-regulation of NADPH oxidase activity (Figure 5).

## Discussion

Diabetic cardiomyopathy is defined as ventricular dysfunction independent of hypertension and coronary artery disease [22]. Apoptotic cell death is increased in the diabetic heart of patients and animal models [6,23] and promotes cardiomyopathy [6]. The continuous loss of cardiomyocytes triggers myocyte hypertrophy and fibrosis, two general hallmarks of diabetic cardiomyopathy [7]. while the mechanism of hyperglycemia-induced apoptosis is poorly understood, cell death by apoptosis is reportedly the predominant damage in diabetic cardiomyopathy [6]. Moreover, diabetes increases cardiac apoptosis in animals and patients [6,7,23]. TLRs play a vital role in host defense but have also been described as a promoter of apoptosis in myocardial ischemia and dysfunction studies. Of the 10 TLRs identified in humans, as least two, TLR2 and TLR4, exist abundantly in the heart [24]. However, the role of TLR4 in enhancing apoptosis of cardiomyocytes induced by hyperglycemia has not been characterized. In this study, we demonstrate that hyperglycemia can trigger cell death pathways in myocardial tissues. For instance, we observed elevations in the apoptotic gene Fas as well as increased activation of apoptotic caspases, such as caspase-3 in diabetic hearts. In addition, we demonstrate that TLR4 is significantly increased in the myocardia of STZ-treated mice. The apoptosis of cardiomyocytes in a high glucose environment can be attenuated by knockdown of the TLR4 gene. Furthermore, apoptosis is associated with increased ROS production and up-regulation of NADPH oxidase activity in diabetic hearts.

TLRs recognize specific structures of microorganisms (pathogen-associated molecular patterns or PAMPs), as well as injury-induced host-derived ("self") structures (damage-associated molecular patterns, or DAMPs) [25]. Upon recognition of PAMPs and DAMPs through direct interaction and signal transduction, TLRs activate various intracellular signaling adaptors. The signaling of TLRs occurs in the cytoplasmic portion of TLR, which shows great similarity to that of the IL-1 receptor family and is termed Toll/IL-1 (TIL) domain. All TLRs possess a cytoplasmic toll IL-1 receptor (TIR) domain, and most activated signaling cascades occur through two pathways: MyD88/NF-kB [26] and TRIF/IRF-3 [27]. Most TLRs utilize the MyD88/NF-kB pathway that is essential for induction of inflammatory cytokines such as TNF-α and IL-1. A few TLRs (eg., TLR3 and TLR4) can activate alternative TRIF/IRF-3, which results in the induction of type I interferons (IFNs) [28]. Therefore, in terms of apoptosis, activation of TLRs in the myocardia may initiate either pro-apoptotic or anti-apoptotic mechanisms [24,29].

Activation of TLR4 may trigger expression of cell survival and inflammatory genes via NF-B-dependent mechanisms. Sustained lipopolysaccharide (LPS, the ligand of TLR4) treatment in rat hearts initiated pro-apoptotic and survival pathways. In the same study, cardiomyocyte apoptosis was minor after LPS treatment [30]. Interestingly, this modest level of apoptosis cannot be responsible for LPS-induced cardiomyocyte dysfunction and thus, the importance of this observation is difficult to ascertain. Furthermore, a recent study indicated that apoptosis resulting from myocardial ischemia-reperfusion injury was decreased upon *in vivo *administration of LPS [31]. After LPS administration, apoptosis did not occur except in cases where endogenous survival protein synthesis was blocked [32], thus providing further indication of parallel survival pathways in endothelial and similar cell types. It is likely that TLR4 and MyD88 cooperatively mediate the anti-apoptotic effect seen in cardiomyocytes after LPS administration [33]. In this study, we demonstrated an up-regulation of TLR4-induced apoptosis in diabetic hearts.

Diabetic hearts generally have ROS levels that exceed normal amounts and likely contribute to cardiomyopathy. ROS production may be enhanced by hyperglycemia in cardiomyocytes [19,23]. Treatment with antioxidants can protect cardiomyocytes from apoptosis in high glucose conditions and as such ROS are thought to play a key role in cardiomyocyte apoptosis in diabetes [6,23]. The pathways culminating in accelerated ROS production and the influence of hyperglycemia on said pathways require further study, however, multiple sources of ROS have been proposed including NADPH oxidase. NADPH oxidase activity, an important factor in the maintenance of the myocardial redox state, is elevated in diabetes [17,20] and can also be over-activated by exposure to high glucose [21]. In the present study, ROS production and NADPH oxidase activity are significantly increased in diabetic mice yet both are suppressed by the knockdown of TLR4 siRNA. Taken together, our data suggests hyperglycemia in diabetic mice may first up-regulate NADPH oxidase, which subsequently increases ROS products which are recognized as harmful by TLR4. In support of this view, our previous study has shown that activation of TLR4 induces NADPH oxidase activation and ROS production in cardiomyocytes [15]. The activation of TLR4 and it's down-stream signaling pathways lead to up-regulation of TNF and IFN [34], which stimulate apoptotic caspase signaling and result in the apoptosis of cardiomyocytes.

Finally, we explored the therapeutic intervention of apoptosis using siRNA. Specific silencing of genes with siRNA is an advanced method of RNA interference [35] that is more potent and specific in the knockdown of gene expression than conventional blocking methods [36,37]. In this study, we used siRNA to knock down TLR4 gene and showed that the use of TLR4 siRNA can prevent myocardial apoptosis in STZ mice, thus highlighting the potential clinical use of siRNA-based therapy.

## Conclusion

In summary, this study defined the role of TLR4 in hyperglycemia-induced apoptosis in STZ mice. Treatment with TLR4 siRNA prevented hyperglycemia-induced apoptosis, highlighting a novel RNAi-based therapy for diabetic cardiac complications using TLR4 siRNA.

## Abbreviations

siRNA: small interfering RNA; TLR: Toll-like receptor: STZ: streptozotocin; ROS: reactive oxygen species.

## Competing interests

The authors declare that they have no competing interests.

## Authors' contributions

YZ, HZ, XiZ, XuZ, NJ, AS, carried out the experiments, WM, NT, TP, YZ, MS, XC, XL, NR, VB participated in the project design, coordination the experiments, and helped to draft the manuscript. All authors read and approved the final manuscript.
